# *TIAM2* Contributes to Osimertinib Resistance, Cell Motility, and Tumor-Associated Macrophage M2-like Polarization in Lung Adenocarcinoma

**DOI:** 10.3390/ijms231810415

**Published:** 2022-09-08

**Authors:** Lu Liang, Hua He, Shiyao Jiang, Yueying Liu, Jingjing Huang, Xiaoyan Sun, Yi Li, Yiqun Jiang, Li Cong

**Affiliations:** 1 The Key Laboratory of Model Animal and Stem Cell Biology in Hunan Province, Hunan Normal University, Changsha 410013, China; 2 School of Medicine, Hunan Normal University, Changsha 410013, China

**Keywords:** lung adenocarcinoma, lipid metabolism, osimertinib resistance index, M2-like tumor-associated macrophage, *TIAM2*

## Abstract

**Background:** Osimertinib-based therapy effectively improves the prognosis of lung adenocarcinoma (LUAD) patients with epidermal growth factor receptor mutations. However, patients will have cancer progression after approximately one year due to the occurrence of drug resistance. Extensive evidence has revealed that lipid metabolism and tumor-associated macrophage (TAM) are associated with drug resistance, which deserves further exploration. **Methods:** An osimertinib resistance index (ORi) was built to investigate the link between lipid metabolism and osimertinib resistance. The ORi was constructed and validated using TCGA and GEO data, and the relationship between ORi and immune infiltration was discussed. Weighted gene co-expression network analysis based on the M2/M1 macrophage ratio determined the hub gene *TIAM2* and the biological function of *TIAM2* in LUAD was verified in vitro. **Results:** ORi based on nine lipid metabolism-related genes was successfully constructed, which could accurately reflect the resistance of LUAD patients to osimertinib, predict the prognosis, and correlate with M2-like TAM. Additionally, *TIAM2* was found to increase osimertinib tolerance, enhance cell motility, and promote M2-like TAM polarization in LUAD. **Conclusions:** The lipid metabolism gene is strongly connected with osimertinib resistance. *TIAM2* contributes to osimertinib resistance, enhances cell motility, and induces M2-like TAM polarization in LUAD.

## 1. Introduction

Lung cancer (LC) is one of the most common types of cancer and Global Cancer Statistics 2020 shows that LC deaths account for 18% of all cancer-related mortality in the world, 85% of which are non-small cell lung cancer (NSCLC) [1,2]. Lung adenocarcinoma (LUAD) is the most common subtype of LC and accounts for more than half of NSCLC [3,4]. LUAD develops rapidly with high malignancy so that by the time of diagnosis, many patients are already in advanced stages of cancer with local invasion (stage III) or distant metastasis (stage IV) of the tumor [5]. Since the prognosis for patients with LUAD who have undergone conventional combination therapy remains unsatisfactory, the emergence of molecularly targeted therapy brings new prospects [6,7,8].

Epidermal growth factor receptor (EGFR) mutations are common types of mutation in LUAD [9]. As the third-generation EGFR tyrosine kinase inhibitors (TKIs) and the first-line therapy for advanced EGFR-mutated NSCLC [10,11], osimertinib can selectively inhibit EGFR that has activating mutations and/or the T790M resistance mutation for the targeted therapy of LUAD [7,12,13,14]. In clinical trials, patients treated with osimertinib not only have longer progression-free survival but also have a lower rate of serious adverse events compared with first-generation EGFR-TKIs (gefitinib or erlotinib) [13,15]. However, approximately 60% of patients will have disease progression at 9–13 months owing to the occurrence of drug resistance [16,17]. Therefore, it is urgent to study the osimertinib resistant mechanisms and discuss the effective regimen of LUAD to increase drug sensitivity

Acquired resistance mechanisms to osimertinib in patients with LUAD can be divided into EGFR-dependent or EGFR-independent. EGFR-dependent mechanisms are mainly associated with mutations and amplifications of EGFR, whereas independent mechanisms can be acquired through activation of bypass pathway, aberrant downstream signaling, or histological transformation such as epithelial–mesenchymal transition (EMT) [18,19]. Dysregulation of lipid metabolism is one of the most prominent metabolic alterations in cancer [20], and recent studies have confirmed that lipid metabolism is involved in regulating the resistance of patients with LUAD to osimertinib through the EGFR signaling pathway [21,22,23]. Another study proves that lipid metabolism is associated with EMT-related drug resistance processes. Simvastatin could reverse A549T (a drug-resistant variant of the parental non-small-cell lung cancer cell A549) EMT-associated drug resistance by targeting cholesterol metabolism to regulate EMT and TAM repolarization [24]. Thus, lipid metabolism might also improve the therapeutic outcomes in drug-resistant LC through EGFR-independent mechanisms such as EMT. Exploring the mechanism of osimertinib resistance through lipid metabolism-related genes may help to provide an effective strategy for the drug selection of the third-generation EGFR inhibitor osimertinib in LUAD patients.

The tumor microenvironment (TME) is an enduring topic, as its components (immune cells, stromal cells, blood vessels, and extracellular matrix) cross-talk with cancer cells and contribute to tumor initiation, progression, metastasis, and resistance to treatment [25,26]. This complex interaction often involves cellular metabolisms, such as lipid metabolism, glucose metabolism, and amino acid metabolism [27,28,29]. In particular, tumor-associated macrophages (TAMs) are the most abundant stromal cell populations in the TME, including classically activated (antitumor M1 phenotype) and alternatively activated state (pro-tumor M2 phenotype) [26,30,31]. Interestingly, some scholars have found that lipid metabolism is closely related to TAM polarization [32,33]. Liu et al. reported that S100A4 enhanced M2-like TAM polarization by controlling the PPAR-γ-dependent induction of fatty acid oxidation [34]. In addition, Wu et al. propose that lipid droplet-dependent fatty acid metabolism polarizes the monocytes into M2-like TAM [35]. We speculate that lipid metabolism plays an important role in the differentiation of pro-tumor TAMs in the TME.

Given the above background, we decided to develop a prognostic osimertinib resistance index (ORi) associated with lipid metabolism genes based on public databases. This index can not only predict the prognosis of patients with LUAD but can guide patients’ therapy. Additionally, differentially expressed genes (DEGs) in the LUAD-TCGA cohort were screened out by the ORi to analyze the relationship between DEGs and immune infiltration and TAM polarization. Finally, weighted gene co-expression network analysis (WGCNA), an analysis of gene co-expression networks based on gene expression data, identified *TIAM2* as the hub gene related to lipid metabolism. Its effects on TAMs pro-tumor phenotypes, osimertinib resistance, and the biological function in LUAD cells were verified by in vitro experiments. Our results may serve as a potential strategy for predicting the prognosis of LUAD and provide new insights into understanding osimertinib resistance in LUAD patients at the molecular level.

## 2. Results

### 2.1. Lipid Metabolism-Related Genes Were Selected as Candidates for ORi

**Figure 1:**
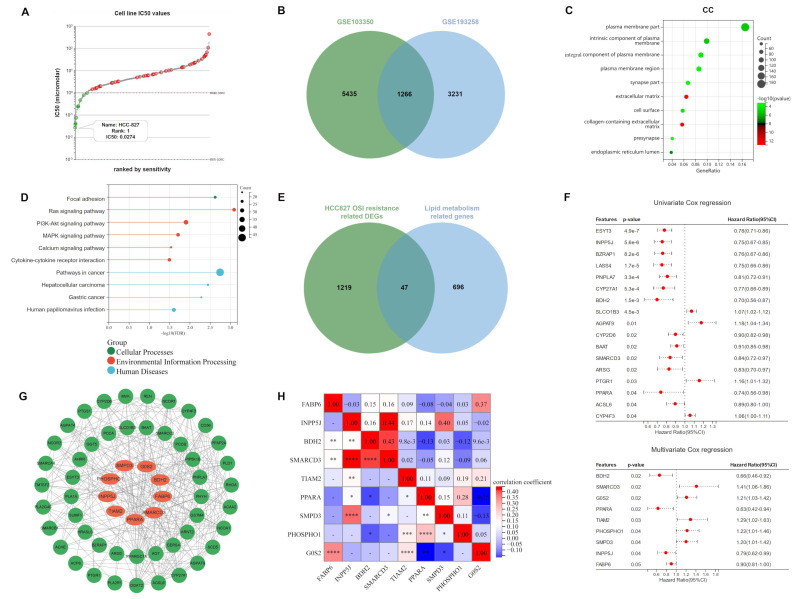
Identification of candidate genes for ORi: (**A**) According to the analysis of the GDSC database, HCC827 has the smallest IC50 value (0.0274 μM) among 62 LUAD cell lines and was considered to be one of the most sensitive to osimertinib. (**B**) DEGs associated with osimertinib resistance in HCC827 at the intersection of GSE103350 and GSE193258. (**C**,**D**) GO and KEGG analyses indicated that these DEGs were enriched in biological processes and pathways related to lipid metabolism. (**E**) Venn diagram showing lipid metabolism-related genes involved in osimertinib resistance. (**F**) Univariate and multivariate Cox regression analysis of 47 lipid metabolism-related genes involved in osimertinib resistance. (**G**) PPI network showing links between 47 proteins. (**H**) Heatmap demonstrating the correlation of 9 candidate genes. ORi: osimertinib resistance index; GDSC: Genomics of Drug Sensitivity in Cancer; LUAD: lung adenocarcinoma; DEGs: differentially expressed genes; GO: Gene ontology; KEGG: Kyoto Encyclopedia of Genes and Genomes; CC: Cellular Component; IC50: half-maximal inhibitory concentration; PPI: protein–protein interactions. HR: hazard rate. * *p* < 0.05; ** *p* < 0.01; *** *p* < 0.001; **** *p* < 0.0001; ns *p* > 0.05.

According to the Genomics of Drug Sensitivity in Cancer (GDSC) database, HCC827 was found to be the most osimertinib-sensitive LUAD cell line, with a half-maximal inhibitory concentration (IC50) of 0.0274 μM (Figure 1A; Table S1). Therefore, we downloaded GSE103350 and GSE193258 from the Gene Expression Omnibus database (GEO, https://www.ncbi.nlm.nih.gov/geo/, accessed on 9 May 2022), and extracted 6701 and 4497 DEGs (FC > 2) related to HCC827 osimertinib resistance, respectively. The intersection of the two datasets contained 1266 DEGs (Figure 1B), for which we performed Gene ontology (GO) and Kyoto Encyclopedia of Genes and Genomes (KEGG) enrichment analyses. Interestingly, as shown in Figure 1C,D and Figure S2, the DEGs involved in HCC827 osimertinib resistance are mainly enriched in lipid metabolism-related pathways and cellular components (CC). Subsequently, we downloaded 743 lipid metabolism-related genes from the Reactome database, 47 of which overlapped with DEGs associated with HCC827 osimertinib resistance. (Figure 1E). Univariate and multivariate Cox regression analyses were performed on 345 samples from the TCGA-LUAD cohort and we obtained 17 (*ESYT3*, *INPP5J*, *BZRAP1*, *LASS4*, *PNPLA7*, *CYP27A1*, *BDH2*, *SLCO1B3*, *AGPAT9*, *CYP2D6*, *BAAT*, *SMARCD3*, *ARSG*, *PTGR1*, *PPARA*, *ACSL6*, and *CYP4F3*) and 9 (*BDH2*, *SMARCD3*, *G0S2*, *PPARA*, *TIAM2*, *PHOSPHO1*, *SMPD3*, *INPP5J*, and *FABP6*) lipid metabolism associated genes with *p* < 0.05, respectively. In addition, *SLCO1B3*, *AGPAT9*, *PTGR1*, *CYP4F3*, *SMARCD3*, *G0S2*, *TIAM2*, *PHOSPHO1,* and *SMPD3* had hazard ratios of more than one, meaning that they are risk genes (Figure 1F). Then, the Protein–Protein Interaction (PPI) network was constructed to predict the interaction of proteins encoded by 47 genes and the network showed that 9 genes in the multivariate Cox regression analysis had significant interactions with other proteins (Figure 1G). Combined with the above analyses, we included *BDH2*, *SMARCD3*, *G0S2*, *PPARA*, *TIAM2*, *PHOSPHO1*, *SMPD3*, *INPP5J,* and *FABP6* as candidate genes for ORi, and visualized their correlations through heat maps (Figure 1H).

### 2.2. Construction and Validation of Ori

**Figure 2:**
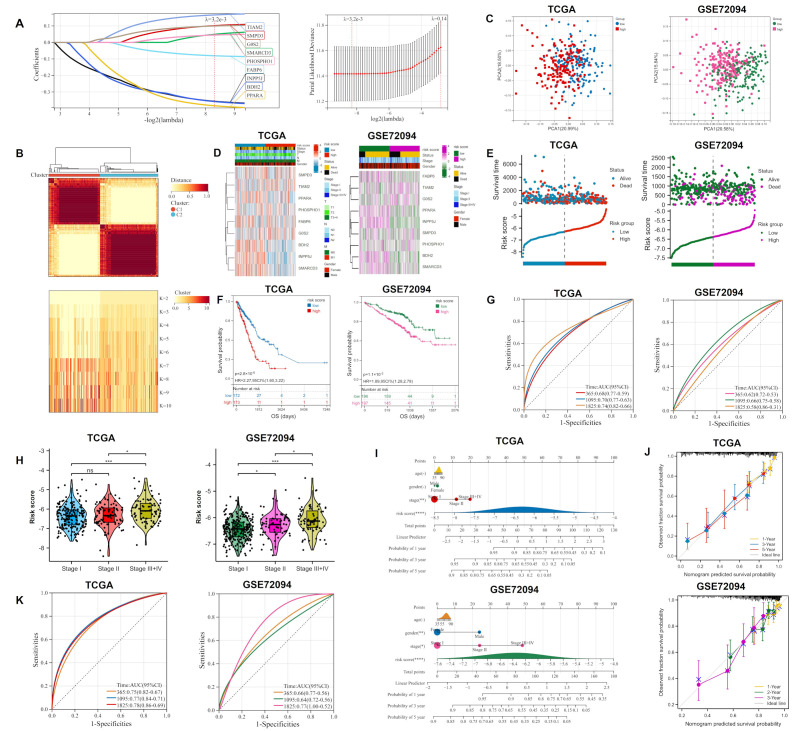
Construction and validation of the ORi composed of 9 lipid metabolism-related genes: (**A**) Nine prognostic genes screened by LASSO regression analysis were used to construct the risk model. (**B**) Consensus clustering determined the best grouping to be 2. (**C**) PCA analysis of grouped samples from the training and validation cohorts. (**D**) Heat map showing the expression of 9 candidate genes grouped according to risk score and other clinical information. (**E**) Risk score distribution and survival status of LUAD patients in high- and low-risk groups. (**F**) KM curves for the two groups reflected the overall survival of patients. (**G**) Time-dependent ROC curve for 365, 1095, and 1825 days based on the risk score. (**H**) Risk score distribution of LUAD patients in different clinical stage groups (stage I, II, and III + IV). (**I**) Nomogram predicting survival probability at 1, 3, and 5 years for LUAD patients (**J**) Calibration curves constructed for the training and validation cohorts validate the accuracy of the nomogram in predicting the prognosis of LUAD patients. (**K**) ROC curve for 365, 1095, and 1825 days based on risk score and other clinical information. LASSO: logistic least absolute shrinkage and selection operator; PCA: principal component analysis; KM: Kaplan–Meier; OS: overall survival; HR: hazard rate; CI: confidence interval; ROC: The receiver operating characteristic. * *p* < 0.05; ** *p* < 0.01; *** *p* < 0.001; **** *p* < 0.0001; ns *p* > 0.05.

The logistic least absolute shrinkage and selection operator (LASSO) identified nine candidate genes (*BDH2*, *SMARCD3*, *G0S2*, *PPARA*, *TIAM2*, *PHOSPHO1*, *SMPD3*, *INPP5J,* and *FABP6*) all relevant to prognosis (Figure 2A). The nine lipid metabolism-related genes mentioned above were used to develop the ORi, and their LASSO regression coefficients are shown in Table S2. Consensus clustering indicated that it was most stable when the grouping was two (Figure 2B). After excluding patients with incomplete clinical information, 345 LUAD samples from The Cancer Genome Atlas (TCGA) were used as a training cohort, which was divided into high- and low-risk groups based on the median risk score. To confirm the results of the analysis of the TCGA cohort, the 394 samples of the validation cohort GSE72094 (with the characteristics of a large sample size and relatively complete clinical information) were also executed synchronously. The principal component analysis (PCA) and T-distribution stochastic neighbor embedding (t-SNE) are two algorithms used to downscale the data and measure their variability. The majority of the patient samples in the high and low-risk groups were found to be projected in different regions of the lower dimension, which reveals the differences in patient features between the two groups (Figure 2C and Figure S3A,B). The expression of nine lipid metabolism-related genes in high and low-risk groups was presented in the heatmap (Figure 2D). It could be seen that *SMARCD3*, *G0S2*, *PPARA*, *TIAM2*, *PHOSPHO1*, and *SMPD3* were highly expressed in the high-risk group compared with the other group. This was consistent with multivariate Cox regression analysis and also implied that the six lipid metabolism-related genes were probably the risk genes involved in osimertinib resistance in LUAD. Furthermore, both the risk factor graph (Figure 2E) and Kaplan–Meier (KM) survival curves (Figure 2F) showed a worse prognosis for patients in the high-risk group of the ORi. A time-dependent receiver operating characteristic (ROC) curve confirmed the reliability of the ORi to predict the 1-, 3-, and 5-year prognosis of LUAD patients (Figure 2G). Samples were divided into three groups (stage I, II, and III+IV) depending on the clinical stage of the LUAD patient and we found that patients with a higher clinical stage have higher risk scores (Figure 2H). Moreover, we built nomograms that enabled the calculation of LUAD patients’ prognosis at 1, 3, and 5 years based on risk score and clinical characteristics (including age, gender, and stage) (Figure 2I). In the nomogram calibration curve, the survival prediction curve is close to the ideal curve (slope is one), indicating the smaller deviation of the nomogram prediction results from the actual survival probability (Figure 2J). In the training cohort, the ROC curves combining risk scores and clinical characteristics achieved an area under the curve (AUC) of 0.75, 0.77, and 0.78 in 1, 3, and 5 years, respectively. The AUC reached 0.66, 0.64, and 0.77 in 1, 3, and 5 years, respectively, by external validation of the GSE72094 cohort. These results illustrated the good accuracy of ORi for predicting the survival of LUAD patients.

### 2.3. ORi Was Associated with M2-like TAM Infiltration

**Figure 3:**
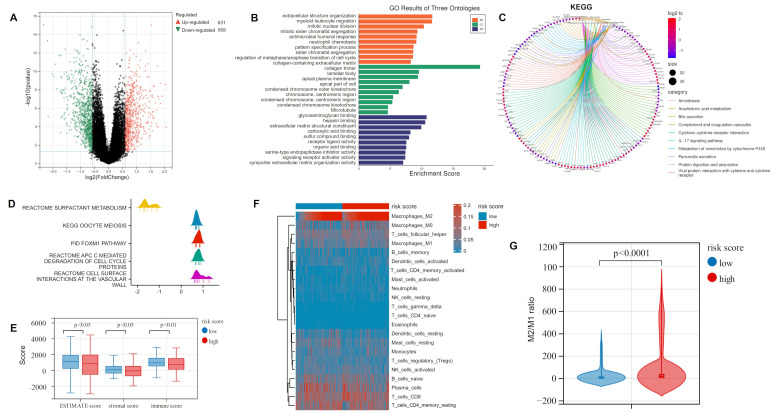
Functional enrichment and immune infiltration analysis of DEGs in high- and low-risk groups: (**A**) Volcano plot showing the DEGs in the TCGA-LUAD cohort based on the median risk score. (**B**) GO analysis revealed that DEGs were mainly focused on immune-related biological processes. (**C**) KEGG analysis indicated that DEGs were mainly concentrated in immune-related pathways. (**D**) GSEA analysis of DEGs. (**E**) Comparison of ESTIMATE score, stromal score, and immune score between the two groups. (**F**) CIBERSORT algorithm assessing 22 types of immune cell infiltration. (**G**) M2/M1 ratio in the high- and low-risk groups. DEGs: differentially expressed genes; GO: Gene ontology; KEGG: Kyoto Encyclopedia of Genes and Genomes; GSEA: Gene Set Enrichment Analysis.

Differential expression analysis was conducted in the high-risk and low-risk groups of the training cohort. Then, 631 up-regulated and 858 down-regulated DEGs were obtained with FC > 1.5 and *p* < 0.05 (Figure 3A; Table S3). The following GO and KEGG analysis found that the majority of these DEGs were enriched in immune-related biological processes (BP) and pathways, such as myeloid leukocyte migration, antimicrobial humoral response, and complement and coagulation cascades (Figure 3B,C). Gene Set Enrichment Analysis (GSEA) analysis found DEGs concentrated on reactive surfactant metabolism, while studies showed a considerable role for surfactant proteins in innate immunity [36] (Figure 3D). Therefore, we evaluated tumor purity and immune cell infiltration levels in the high and low-risk groups of the training cohort by the ESTIMATE and CIBERSORT algorithms (Figure 3E,F). The above analysis indicated that the high-risk group had a lower ESTIMATE score, stromal score, and immune score than the low-risk group. In the assessment of 22 immune cell infiltrates, we were surprised to find that the level of M2 TAM infiltration was not only higher than in other immune cells in LUAD patients but also significantly different between high- and low-risk groups. High TAM infiltration is often associated with poor prognosis in most tumors (including LC), and M2 TAM, as a major component of TAMs, presented pro-tumor efficacy [37]. We further calculated the M2/M1 ratio of LUAD samples. As shown in Figure 3G, the lower risk group has a lower M2/M1 ratio than the other group, suggesting that LUAD patients who are more resistant to osimertinib have higher levels of M2 infiltration.

### 2.4. TIAM2 Involved in Immune Infiltration and Osimertinib Resistance

**Figure 4:**
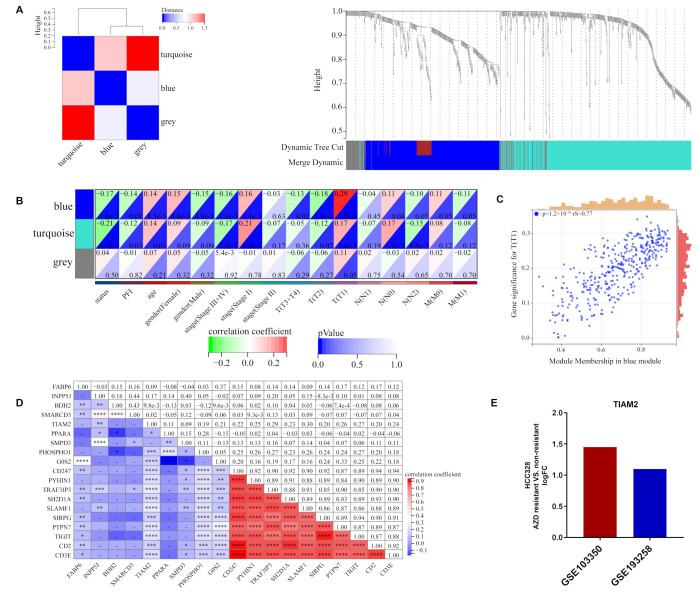
WGCNA analysis of DEGs in high and low M2/M1 groups: (**A**) Clustering of DEGs based on M2/M1 ratio. (**B**) Heat map of correlations between individual gene modules and traits. (**C**) The scatter plot suggests that the blue module had a strong connection to clinical stage T1 (*p* < 0.05, rS = 0.77). (**D**) Correlation analysis of 9 ORi’s candidate genes and blue module top 10 genes. (**E**) LogFC of *TIAM2* expression in osimertinib resistant and non-resistant HCC827 for GSE103350 and GSE193258, respectively. WGCNA: weighted gene co-expression network analysis; DEGs: differentially expressed genes. * *p* < 0.05; ** *p* < 0.01; *** *p* < 0.001; **** *p <* 0.0001; ns *p* > 0.05.

Based on the M2/M1 ratio, 1002 DEGs were acquired from the training cohort, with 327 up-regulated and 675 down-regulated genes (Figure S4A, FC > 1.5, *p* < 0.05). Then, these DEGs were divided into three modules (turquoise, blue, and gray) by WGCNA analysis according to different expression patterns (Figure 4A). The heatmap of module-phenotype correlation showed that the blue module was significantly associated with clinical stage T1 (Figure 4B) and the scatter plot of blue module members related to T1 as depicted in Figure 4C and Table S4 (*p* < 0.05, rS = 0.77). Further, GO and KEGG enrichment analysis revealed that DEGs were primarily participating in lipid-related cellular composition (CC) and drug resistance-related pathways such as the integral component of the membrane and the nuclear factor-kB (NF-kB) signaling pathway (Figure S5A,B and Table S5). We performed a correlation analysis of the top 10 blue module members with the nine candidate genes of the ORi in the TCGA-LUAD cohort (Figure 4D). Both *TIAM2* and *PHOSPHO1* showed a stronger correlation with blue module members compared to the other seven candidate genes, with *p* < 0.001. Combined with the LASSO regression coefficients, we selected the lipid metabolism-related gene *TIAM2* as the hub gene involved in osimertinib resistance and immune regulation. Finally, we concluded that the logFC of GSE103350 and GSE193258 reached 1.45 and 1.10, respectively, by comparing the expression of *TIAM2* in osimertinib resistant and non-resistant HCC827 (Figure 4E). *TIAM2* is a lipid metabolism-related gene involved in immune regulation and drug resistance, and the potential link between its expression and osimertinib tolerate and resistant patient needs to be further explored.

### 2.5. TIAM2 Correlated with Prognostic, Immunity, Mutational, and Pathological Changes in LUAD Patients

**Figure 5:**
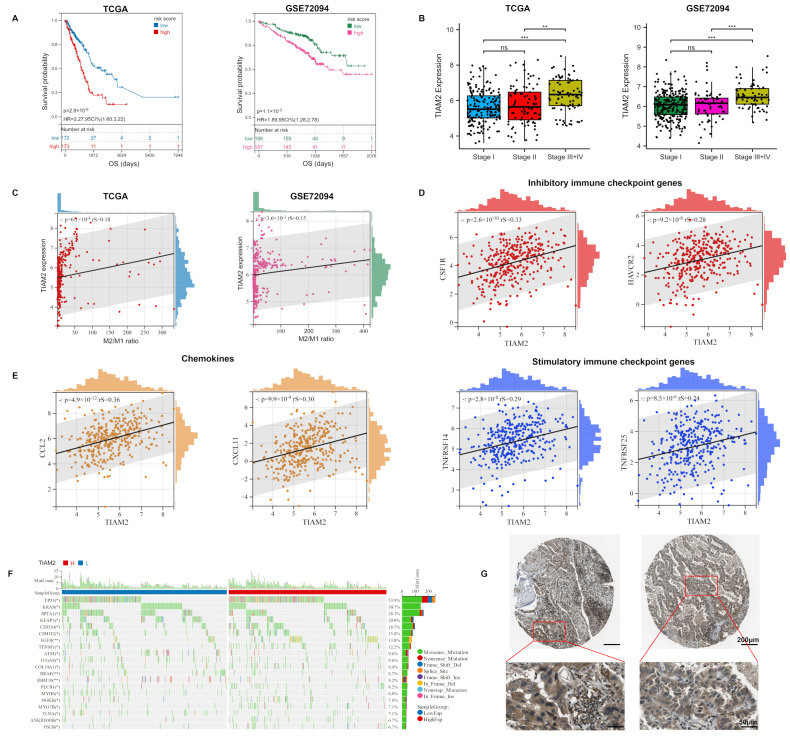
Expression of *TIAM2* correlated with prognostic, immunity, mutational, and pathological changes in LUAD patients: (**A**) KM curves showed that high expression of *TIAM2* was associated with poor survival of LUAD patients. (**B**) The *TIAM2* expression level of LUAD patients in different clinical stage groups (stage I, II, III+IV). (**C**) Correlation between M2/M1 ratio and *TIAM2*. (**D**) Expression correlation analysis between immune checkpoint genes (*CSF1R*, *HAVCR2*, *TNFRSF14,* and *TNFRSF25*) and *TIAM2*. (**E**) Expression correlation analysis between chemokines (CCL2 and CXCL11) and *TIAM2*. (**F**) Mutational landscape of *TIAM2* high- and low-expressing groups. (**G**) Two LUAD samples of TIAM2 protein expressions were observed by HPA. OS: overall survival; HR: hazard rate; CI: confidence interval; HPA: Human Protein Atlas. ** *p* < 0.01; *** *p* < 0.001; ns *p* > 0.05.

We further analyzed the LUAD samples based on the expression of *TIAM2*. The survival analysis results of both cohorts suggested that the low expression group of *TIAM2* had a better survival probability than the high expression group (Figure 5A, *p* < 0.05). In the samples grouped by different clinical stages, the expression of *TIAM2* was significantly higher in the group with stages III + IV than in the others (stage I, stage II). (Figure 5B). In addition, *TIAM2* expression was positively correlated with the M2/M1 ratio in the training and validation cohorts (Figure 5C, *p* < 0.01). In clinical practice, the application of immune checkpoint blockade therapy could benefit patients with NSCLC [38]. Immune checkpoint genes encode immune checkpoint molecules that regulate the level of immune activation. Subsequently, we noticed that in the TCGA-LUAD cohort, the expression of inhibitory immune checkpoint genes *CSF1R* and *HAVCR2* and stimulatory immune checkpoint genes *TNFRSF14* and *TNFRSF25* were positively correlated with the risk gene *TIAM2* (Figure 5D). It has been reported that cancer cells can evade immune surveillance by up-regulating inhibitory immune checkpoint genes during tumorigenesis [39], which is consistent with our above result. In addition, high expression of *TNFRSF14* and *TNFRSF25* has also been proven to be relevant to the poor prognosis of LC patients [40,41]. Chemokines also play an essential role in the regulation of tumor immunity. In Figure 5E, we can observe that the expression of two pro-carcinogenic chemokines CCL2 and CXCL11 increases with the *TIAM2* expression [42,43]. We further compared the gene mutation frequencies in high and low *TIAM2* expression groups and displayed the top 20 genes (Figure 5F). Our results showed that *EGFR* was the seventh most frequent mutation in the TCGA-LUAD sample and was much more frequent in the *TIAM2* high-expression group than the low-expression group, meaning that LUAD patients with higher levels of *TIAM2* expression are more likely to have mutations in *EGFR* and might be more resistance to osimertinib. We investigated the protein expression pattern of TIAM2 using the Human Protein Atlas (HPA) and discovered that TIAM2 expression was higher in the cytoplasm and membrane of LUAD cells (Figure 5G).

### 2.6. TIAM2 Contributed to Osimertinib Resistance, Cell Motility, and M2-like TAM Polarization in LUAD

**Figure 6:**
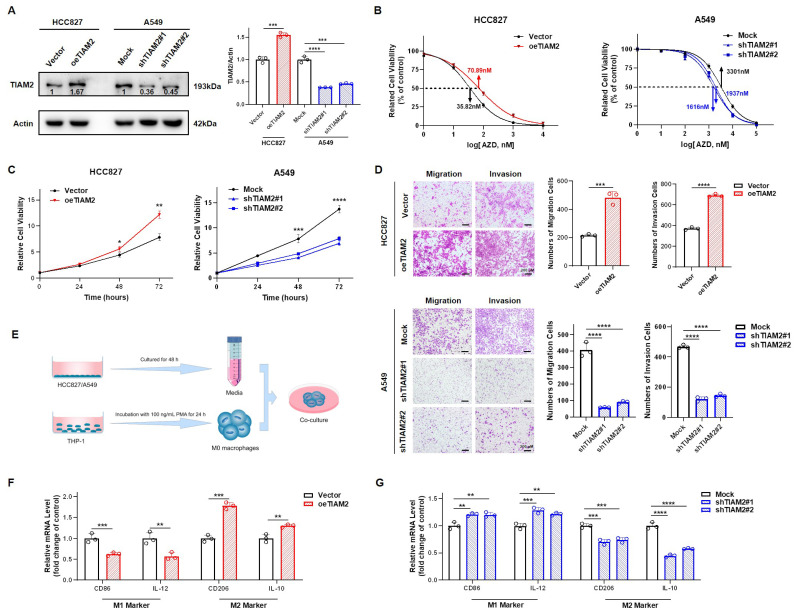
Experimental verification reveals *TIAM2* is involved in osimertinib resistance, cell motility, and macrophage polarization in LUAD: (**A**) Left, validation of the efficacy of *TIAM2* overexpression or knockdown by WB. Right, amounts of TIAM2 were quantified from the experiment. (**B**) CCK-8 assay suggested that knockdown of *TIAM2* reduced the resistance of LUAD cells to osimertinib, whereas overexpression did the opposite. (**C**) The proliferation of HCC827 overexpressing *TIAM2* was significantly enhanced, while the proliferation of A549 with *TIAM2* knocked down was significantly inhibited. (**D**) The migration and invasion abilities of HCC827 overexpression *TIAM2* were significantly enhanced, and A549 knockdown *TIAM2* was significantly weakened. (**E**) Schematic diagram of conditioned medium treatment. (**F**,**G**) The supernatant of HCC827/A549 was used for M0 macrophage culture, and the mRNA levels of M1/M2 marker genes were detected by RT-qPCR after 48 h. HCC827: a type of LUAD cells sensitive to osimertinib; A549: a type of LUAD cells relatively resistant to osimertinib; oe*TIAM2*: overexpression *TIAM2*; Mock: transferred into a scramble control vector; sh*TIAM2*#1: transferred into Number 1 short hairpin RNA (shRNA) that interferes with *TIAM2* expression; sh*TIAM2*#2: transferred into Number 2 shRNA that interferes with *TIAM2* expression; n = 3. * *p* < 0.05; ** *p* < 0.01; *** *p* < 0.001; **** *p* < 0.0001; ns *p* > 0.05.

We chose to overexpress and knock down the lipid metabolism-related gene *TIAM2* in HCC827 and A549 respectively and verified by Western blot (WB, Figure 6A). The mRNA expression levels of *SMARCD3*, *G0S2*, *PPARA*, *TIAM2*, *PHOSPHO1*, and *SMPD3* (hazard rate: HR > 1 in multivariate Cox regression analysis) in overexpressed and knockdown cell lines were detected by Real-Time Quantitative PCR (RT-qPCR). We found that the expression level of *G0S2* and *PPARA* were consistent with the changes in *TIAM2*, but *SMARCD3*, *PHOSPHO1,* and *SMPD3* did not produce significant variation in the overexpressed or knockdown cell lines(Figure S6). Additionally, GSVA analysis revealed differences in the enrichment of the PI3K/Akt/mTOR signaling pathway between high and low-risk groups of the model (Figure S7). We examined the protein expression levels of p-EGFR, EGFR, p-AKT, and AKT, in both overexpressed and knockdown cell lines by WB. As shown in Figure S8, p-EGFR, and p-AKT are upregulated in response to overexpression of *TIAM2* and downregulated in response to the silencing of *TIAM2*. The IC50 values of HCC827 and A549 for osimertinib and their proliferative capacity were tested by the CCK-8 kit. As shown in Figure 6B, the IC50 value of *TIAM2*-overexpressing HCC827 is 70.89 nM, 35.07 more than the 35.82 nM of the vector control. Instead, the IC50 value of sh*TIAM2*#1, and #2 of A549 is 1616 nM and 1936 nM, 1665 and 1365 less than the 3301 nM of the Mock group. We observed the proliferation of cells within 72 h and found that compared to the control group, the proliferation of HCC827 overexpressing *TIAM2* was significantly enhanced, while the proliferation of A549 with *TIAM2* knocked down was significantly inhibited (Figure 6C). Then, we used the transwell assay to explore the influence of *TIAM2* expression on the migration and invasion ability of LUAD cell lines. The results of the statistical analysis showed that the migration and invasion abilities of the *TIAM2* stable overexpression cell line were significantly enhanced, and the stable knockdown *TIAM2* cell line was weakened (Figure 6D). The process of conditioned medium treatment is shown in Figure 6E. We collected supernatants from HCC827/A549 cells with/without the interference of *TIAM2* expression, and then incubated M0 macrophages with culture supernatants from different sources. Relative expression of M1 (*CD86*, *IL-12*) and M2 (*CD206*, *IL-10*) genes were detected by RT-qPCR (Figure 6F,G). Compared to the vector group, macrophages treated with medium from HCC827 overexpressing *TIAM2* were found to have reduced expression of M1 marker genes CD86 and *IL-12*, while M2 macrophage marker genes *CD206* and *IL-10* were strongly upregulated. Thus, we conclude that *TIAM2* overexpression can promote the polarization of M2-like TAM. Moreover, *TIAM2* knockdown suppressed the polarization of M2-like TAM compared with the mock group. Overall, the lipid metabolism-related gene *TIAM2* is not only involved in osimertinib resistance, invasion, and migration of cancer cells but also contributes to M2-like TAM polarization in LUAD.

## 3. Discussion

For the past ten years, EGFR TKIs targeting EGFR mutations have brought remarkable efficacy to LC patients. However, the development of acquired drug resistance has prevented a durable response to their treatment [44]. Osimertinib is a third-generation EGFR TKI that is not only a first-line therapy for targeting EGFR mutations in NSCLC but also a second-line treatment for first/second-generation EGFR TKIs resistance [45]. Compared to the first/second-generation EGFR TKIs, osimertinib has a much more complex resistance mechanism and is still not fully elucidated [46]. At present, scholars are working to explore EGFR-independent mechanisms such as bypass pathways [47], in addition to EGFR-dependent mechanisms [48]. Perhaps, by analyzing RNA-sequencing data and clinical data related to osimertinib resistance, new methods and insights can be provided to guide the drug therapy of osimertinib-resistant LUAD patients.

Studies have found that lipid metabolism within the TME plays an essential role in cancer [49,50]. M2 macrophage polarization is dependent on lipid metabolism for energy [51], and not only that, membrane cholesterol efflux and lipid raft depletion of TAM can mediate TME reprogramming [52]. Abnormal expression of genes related to lipid metabolism is also associated with metastasis, drug resistance, and relapse of cancer [53]. For example, deficiency of *RIPK3* in hepatocellular carcinoma promotes TAMs infiltration levels and M2-like TAM polarization through the lipid metabolism pathway in TME [54]. There is evidence that the lipid signaling molecule 14, 15-epoxyeicosatrienoic acid induces breast cancer invasion, metastasis, and cisplatin resistance [55]. Sterol regulatory element-binding protein 1 (*SREBP1*) is an important gene in the regulation of lipid metabolism, and Chen et al. demonstrated that elevated expression of *SREBP1* is directly related to acquired resistance to osimertinib NSCLC [22]. In this work, we identified nine lipid metabolism-related genes involved in osimertinib resistance in LUAD.

The lipid metabolism gene set was obtained based on the Reactome Pathway database, which is supported by the literature. Through bioinformatics methods, we successfully developed an osimertinib-related risk index based on nine lipid metabolism-related genes and named it the ORi. LUAD patients were grouped by calculated risk score, with patients in the high-risk group representing high resistance to osimertinib and those in the low-risk group showing the opposite. Therefore, we could assess whether to use osimertinib as a front option for individualized treatment based on ORi. Moreover, the nomogram with ORi enables the calculation of the survival probability at 1, 3, and 5 years for each sample. Results of relevant bioinformatic analysis demonstrated ORi has strong predictive power for the probability of survival of LUAD patients.

Functional enrichment analysis suggested that DEGs in the high- and low-risk groups were involved in immune-related processes and pathways. Subsequent immune infiltration analysis revealed that the expression of M2-like TAM was pretty high in TCGA-LUAD samples and that there were differences between the two groups. To investigate the role of M2-like TAM in promoting osimertinib resistance to LUAD, we further clustered TCGA-LUAD samples by median M2/M1 ratio. DEGs in the high M2/M1 ratio and low M2/M1 ratio groups were enriched in lipid-related CC and drug resistance-related pathways. Studies have reported that the NF-kB signaling pathway is not only involved in the regulation of immunity [56], but its activation may also lead to osimertinib resistance [57]. Finally, we determined that the lipid metabolism-related gene *TIAM2* was a hub gene involved in osimertinib resistance and M2 TAM infiltration in ORi through the WGCNA algorithm.

The lipid metabolism-related gene *TIAM2*, a rac1-specific guanine nucleotide exchange factor (GEFs), can activate GTPases by facilitating binding to GTP [58,59], and is highly concentrated in lipid rafts [60]. *TIAM2* has been reported to regulate cell movement in a specific direction by focal adhesion disassembly. However, motility disorders may contribute to the initiation and metastases of tumors [61]. Previous studies have also shown that *TIAM2* enhances the proliferation, migration, and invasion of LC cells as *TIAM2* is not only an important receptor tyrosine kinase effector for Rac1-dependent cell motility [62], but also can affect cancer cells via cancer-associated fibroblasts [63]. In this study, we found that high *TIAM2* expression predicted a worse prognosis and a high M2/M1 ratio. Coincidentally, several studies have shown that immune checkpoint genes *CSF1R* and *HAVCR2* are closely associated with the induction of TAMs polarization toward the M2 phenotype [64,65], and chemokine CCL2 plays a key role in the recruitment of macrophages to neoplastic tissue [66], while our analysis showed a positive association between these genes and *TIAM2*. More interestingly, the EGFR mutation rate was significantly higher in the *TIAM2* high expression group, which indicated that LUAD patients with high expression of *TIAM2* may not be suitable for osimertinib as a first-line treatment option. After further experimental validation, we confirmed that lipid metabolism-related gene *TIAM2* could increase the tolerance of cancer cells to osimertinib, promote cell motility, and induce M2-like TAM polarization in LUAD.

Based on our research and that of other scholars, [67,68], we hypothesize that overexpression of *TIAM2* may mediate osimertinib resistance by activating the PI3K/Akt/mTOR signaling pathway to induce M2-like TAM polarization, and thus there is a potential role for *TIAM2* in tumor immunotherapy and targeted therapy with osimertinib. In addition, combination treatment modalities of immunotherapy and targeted therapy have provided significant survival benefits in the treatment of advanced NSCLC patients [69]. Studies have shown that NSCLC patients with EGFR mutations respond poorly to anti-PD-1/PD-L1 therapy targeting T cells [70]. In such a situation, the combined modality of osimertinib and immunotherapy targeting TAM is still unclear and may be worth further exploration. In other words, immunotherapy against TAM may also be a new choice for LUAD patients who are resistant to osimertinib. For example, the lipid metabolism-related gene *TIAM2* might be able to regulate osimertinib resistance by inducing macrophage phenotypic shifts and remodeling TAMs and TME [71]. However, our research is still inadequate. For the bioinformatics section, it would be advisable to use more clinical data to corroborate the results. In addition, the pathways and mechanisms through which *TIAM2* contributes to the polarization of M2-like TAM and resistance to osimertinib in LUAD remain elusive and need to be further explored.

## 4. Materials and Methods

### 4.1. Collection of Public Data

The osimertinib IC50 values for LUAD cell lines were obtained from GDSC (https://www.cancerrxgene.org/, accessed on 20 May 2022) database. mRNA profiles of osimertinib resistance in HCC827 were derived from GSE103350 and GSE193258. Lipid metabolism-related genes were extracted from Reactome pathway database (https://reactome.org/, accessed on 9 May 2022). Data of LUAD samples from TCGA and GSE72094 were acquired for subsequent analyses after excluding patients with incomplete clinical information. The flowchart of this study is shown in Figure S1.

### 4.2. Enrichment Analysis

GO, KEGG enrichment analysis, and GSEA were performed on DEGs by R package ‘clusterProfiler’ in this study [72].

### 4.3. Protein-Protein Interactions (PPI) Networks and Correlation Analysis

Lipid metabolism-related and osimertinib resistance-related genes were imported into the Retrieval of Interacting Genes (STRING, https://cn.string-db.org/, accessed on 19 June 2022) database, while the PPI network was visualized by Cytoscape software (version 3.9.1). In addition, correlation analysis was performed between genes, between *TIAM2* expression and the M2/M1 ratio, as well as *TIAM2* expression and immune molecules.

### 4.4. Construction and Validation of Prognostic Osimertinib Sensitive Risk Model

The candidate genes of ORi were identified by LASSO [73] analysis and consensus clustering divided the cohort into two groups. The risk score for each patient was calculated as follows: risk score = sum (each candidate gene expression × corresponding LASSO regression coefficient). The R package ‘ConsensusClusterPlus’ [74] was used to determine the optimal number of subgroups. Subsequently, TCGA-LUAD samples were used as a training cohort and divided into high-risk and low-risk groups based on the median risk score. PCA and t-SNE were used to determine the difference in the grouping. The complex heatmap of candidate genes’ expression in different subgroups (including risk score, status, stage, and gender) was produced by the R package ‘ComplexHeatmap’ [75]. Furthermore, the risk scores of the samples grouped by clinical stage were displayed in a violin plot by the ‘ggplot2′ R package [76]. Risk factor graph and KM curves [77] were utilized to assess the survival state in high and low-risk groups of the ORi. Then we combined information on age, gender, stage, and risk score to develop a nomogram to predict the prognosis of patients with LUAD. ROC [78] and calibration curves were used to evaluate the predictive power of the risk signature. Simultaneously, we employed GSE72094 as the validation cohort and followed the same approaches as above for the external validation of the ORi.

### 4.5. Immune Infiltration Analysis

We used the R package ‘estimate’ to assess the ESTIMATE score, stromal score, and immune score in high- and low-risk groups of the training cohort [79]. To evaluate the differences in expression of multiple immune cells between the two groups, we applied the CIBERSORT algorithms [80]. Moreover, the ratio of M2/M1 TAM in the high and low-risk groups was visualized in the violin plot.

### 4.6. Weighted Gene Correlation Network Analysis (WGCNA)

The TCGA-LUAD cohort was classified into an M2/M1 high ratio group and low ratio group. The DEGs of the two groups were obtained from it based on limma analysis. Subsequently, by using the ‘WGCNA’ R package [81], the DEGs were divided into different modules according to their correlations. We associated the module with some characteristics of the samples, such as status, age, stage, etc. While the genes in the module most associated with the sample traits will be used for correlation analysis with ORi candidate genes.

### 4.7. Genome Mutation and Human Protein Atlas (HPA)

Gene mutant frequency in high- and low- expression groups of *TIAM2* were plotted by the ‘maftools’ package in the R software [82]. Detection of TIAM2 expression at a translational level in lung adenocarcinoma tissue and normal tissue using the HPA database (http://www.proteinatlas.org/, accessed on 23 July 2022) [83].

### 4.8. Cell Culture, Plasmids, and shRNAs

The human acute monocytic leukemia cell line THP-1 (ATCC: TIB-202^TM^) and human LUAD cell lines HCC827 (ATCC: CRL-2868^TM^) and A549 (ATCC: CCL-185^TM^) were acquired from American Type Culture Collection (ATCC). THP-1 and HCC827 were cultured in RPMI 1640 (Gibco, Carlsbad, CA, USA), and A549 was cultured in DMEM/F12 (Gibco, Carlsbad, CA, USA). All cell lines were cultured in a medium supplemented with 10% fetal bovine serum (FBS, Gibco, Carlsbad, CA, USA) and 1% penicillin/streptomycin solution, and maintained at 37 °C with 5% CO_2_. These three cell lines were confirmed to be mycoplasma free and were passaged < 10 times after the initial recovery of the frozen stocks. All cell lines were authenticated by performing short tandem repeat profiling before use.

Human *TIAM2* complement DNA (cDNA) expression vector was constructed by Public Protein/Plasmid Library (Nanjing, China) with pLVX-EF1alpha-IRES-Puro (catalog no. 631988; Clontech, Mountain View, CA, USA).

The shRNA vectors specifically targeting *TIAM2* (sh*TIAM2*#1: 5′-GTTAAGGTGATTCGTTCTATT-3′; sh*TIAM2*#2: 5′-GCCCTACTAAAGACATCGAAA-3′) and a scramble control vector (shGFP: 5′-GCAAGCTGACCCTGAAGTTCA-3′) were purchased from Genechem (Shanghai, China). All the plasmid vectors were verified by performing sequencing [84].

### 4.9. RNA Extraction and Real-Time Quantitative PCR (RT-qPCR)

As previously described [85], RNA was extracted with Trizol reagent (R401-01, Vazyme, Nanjing, China). cDNA synthesis was achieved by HiScript^®^ II Q RT SuperMix for qPCR (+gDNA wiper) (R223-01, Vazyme, Nanjing, China). Real-time quantitative PCR was performed by the Bio-Rad CFX Connect Real-Time PCR System with MonAmp™ ChemoHS qPCR Mix (MQ00401S, Monad, Shanghai, China). The primers for this experiment were purchased from Sangon (Shanghai, China), and the sequences of all primers are listed in Table 1.

### 4.10. Western Blot (WB)

The WB was performed as described before [86]. Proteins of target cells were extracted using a lysate buffer (#87787, Thermo Fisher) containing a protease inhibitor (#P1010, Beyotime). After the protein concentration was determined by BCA assay (#E112-01, Vazyme), 40 μg was used for WB analysis. We purchased the rabbit antibody anti-human *TIAM2* (#ab199426, Abcam), mouse monoclonal antibody anti-human β-actin (#AF7018, Affinity), rabbit antibody anti-human p-EGFR (Tyr 1068) (#AF3045, Affinity), rabbit antibody anti-human EGFR (#AF6043, Affinity), rabbit antibody anti-human AKT1/2/3 (Ser473) (#AF0016, Affinity), rabbit antibody anti-human AKT (#AF6261, Affinity), goat anti-rabbit antibody (#S0001, Affinity), and goat anti-mouse antibody (#A21010, Affinity).

### 4.11. Cell Counting Kit 8 (CCK-8) Assay

The details have been previously described [87]. Both HCC827 and A549 were inoculated at 1 × 10^3^ cells per well in 96-well plates. The viability of the cells treated with CCK-8 reagent (CCK-8, A311-01, Vazyme, Nanjing, China) was measured at 450 nm wavelength after 2 h using a microplate reader (Synergy2, Bio-Tek, Winooski, VT, USA).

### 4.12. Transwell Assay

Transwell assays were performed using transwell chambers with or without Matrigel coating to detect cell invasion and migration ability, respectively. For invasion assay, Matrigel (082706, ABW, China) was dissolved with a serum-free medium at 1:8, and 100 µL was added to each upper chamber. Then, 200 μL serum-free medium containing 5 × 10^4^ was added to the upper chamber, while the lower chamber was added with 600 μL 10% FBS medium. The cells were fixed and stained after 24 h of incubation. For migration assay, 200 μL serum-free medium containing 2.5 × 10^4^ was added to the upper chamber, while the lower chamber was added with 800 μL 10% FBS medium. The next procedures are the same as for the invasion assay. Three randomly selected fields of view were photographed under the microscope and the images were counted using ImageJ software (National Institutes of Health) [88].

### 4.13. Differentiation of THP-1 and Conditioned Medium Treatment

We cultured THP-1 with 100 ng/mL of phorbol 12-myristate 13-acetate (PMA, Sigma) and induced it into M0 macrophages. The supernatant of HCC827 oe*TIAM2* or Vector, A549 sh*TIAM2* or Mock, was subsequently used as the conditioned medium for M0 macrophages for 48 h, then the M1 (*CD86*, *IL-12*) and M2 (*CD206*, *IL-10*) markers were detected by RT-qPCR [89].

### 4.14. Statistical Analysis

All statistical data were analyzed in the R (version 4.2.0) package or GraphPad Prism 8.0. For comparison of the two groups, the Student’s *t*-test was used. Survival analysis was conducted using Cox hazards regression and the KM method in this study. Correlation analysis was performed by Spearman’s test. Each experiment was performed at least 3 times. When *p* < 0.05, the statistical significance of the results was confirmed.

## 5. Conclusions

Collectively, we successfully constructed an osimertinib resistance index based on nine lipid metabolism-associated genes and named it ORi, which can not only accurately predict the prognosis of LUAD patients, but also guide targeted drug selection by assessing patients’ resistance to osimertinib through a risk score. Bioinformatics analysis and in vitro experiments demonstrated that expression of the lipid metabolism-related gene *TIAM2* in LUAD increases the resistance of cancer cells to osimertinib, and cancer cell motility including proliferation, migration, invasion, and drives macrophage M0 to M2 polarization.

## Figures and Tables

**Table 1:** Primers for qPCR.

Primers	Forward	Reverse
CD86	5′-CCATCAGCTTGTCTGTTTCATTCC-3′	5′-GCTGTAATCCAAGGAATGTGGTC-3′
IL-12	5′-CCTTGCACTTCTGAAGAGATTGA-3′	5′-ACAGGGCCATCATAAAAGAGGT-3′
CD206	5′-AGCCAACACCAGCTCCTCAAGA-3′	5′-CAAAACGCTCGCGCATTGTCCA-3′
IL-10	5′-GACTTTAAGGGTTACCTGGGTTG-3′	5′-TCACATGCGCCTTGATGTCTG-3′
β-actin	5′-CACCATTGGCAATGAGCGGTTC-3′	5′-AGGTCTTTGCGGATGTCCACGT-3′

## Data Availability

The data presented in this study are openly available in GEO (https://www.ncbi.nlm.nih.gov/geo/, accessed on 9 May 2022) and TCGA databases (https://portal.gdc.cancer.gov/, accessed on 18 June 2022).

## Supplementary Materials

The following supporting information can be downloaded at: https://www.mdpi.com/article/10.3390/ijms231810415/s1.

## Abbreviations

LC	Lung cancer
NSCLC	non-small cell lung cancer
LUAD	lung adenocarcinoma
ORi	osimertinib resistance index
EGFR	epidermal growth factor receptor
TKIs	tyrosine kinase inhibitors
EMT	epithelial–mesenchymal transition
TME	the tumor microenvironment
TAMs	tumor-associated macrophages
DEGs	differentially expressed genes
WGCNA	weighted gene co-expression network analysis
GDSC	Drug Sensitivity in Cancer
IC50	half-maximal inhibitory concentration
GEO	Gene Expression Omnibus database
GO	Gene ontology
KEGG	Kyoto Encyclopedia of Genes and Genomes
PPI	protein–protein interactions
LASSO	logistic least absolute shrinkage and selection operator
TCGA	The Cancer Genome Atlas
PCA	principal component analysis
t-SNE	T-distribution stochastic neighbor embedding
ROC	The receiver operating characteristic
AUC	area under the curve
KM	Kaplan–Meier
OS	overall survival
HR	hazard rate
CI	confidence interval
BP	biological processes
GSEA	Gene Set Enrichment Analysis
NF-kb	nuclear factor-Kb
CC	cellular composition
GEFs	guanine nucleotide exchange factors
HPA	Human Protein Atlas
WB	Western blot
RT-qPCR	Real-Time Quantitative PCR
shRNA	short hairpin RNA
ATCC	American Type Culture Collection
cDNA	complement DNA
CCK-8	Cell Counting Kit 8
PMA	phorbol 12-myristate 13-acetate
