# Supplementary material for: TIAM2 Contributes to Osimertinib Resistance, Cell Motility, and Tumor-Associated Macrophage M2-like Polarization in Lung Adenocarcinoma
Source: Int J Mol Sci. 2022 Sep 8;23(18):10415. doi: 10.3390/ijms231810415 (PMC9499457; doi:10.3390/ijms231810415)
Supplement: Supplementary file 1 [file ijms-23-10415-s001.zip › Supplementary Figures.pdf]

Supplementary Figure Legends

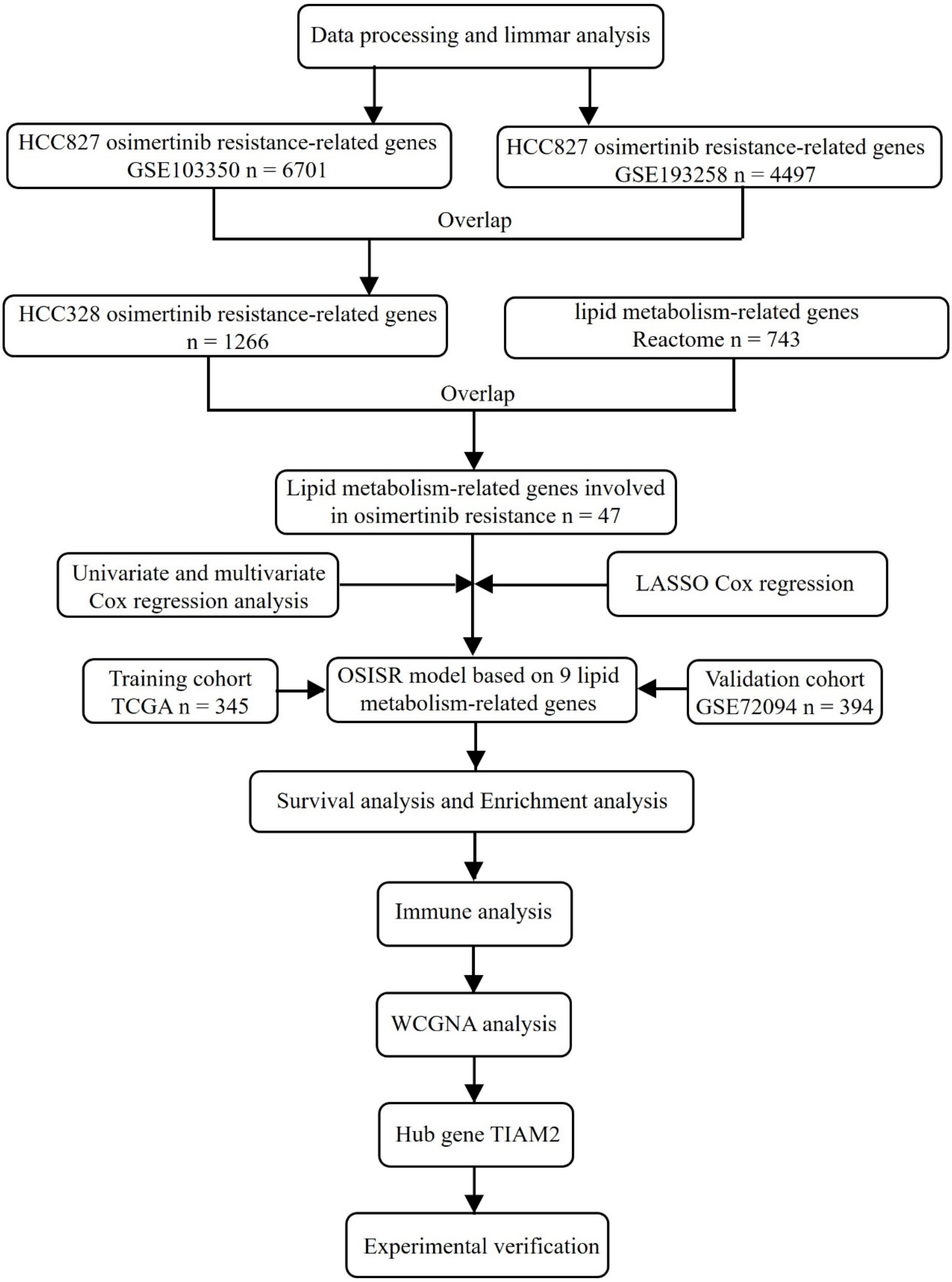

Figure S1 Study flow diagram.

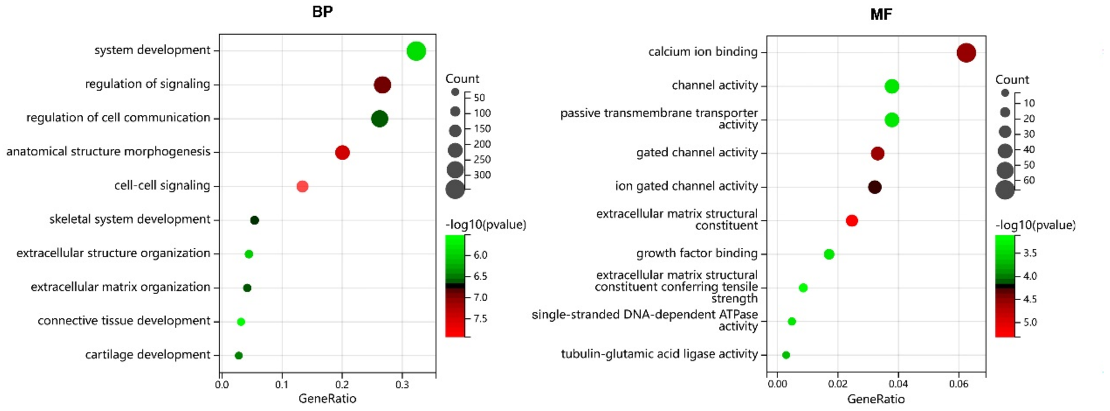

Figure S2 GO enrichment analysis showing the biological processes and molecular functions.

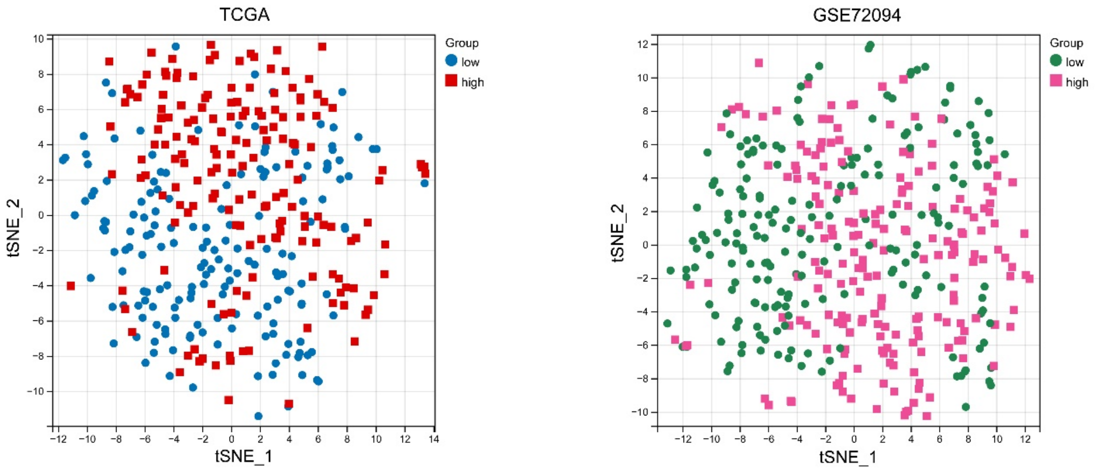

Figure S3 t-SNE analysis of grouped samples from the training and validation cohorts.

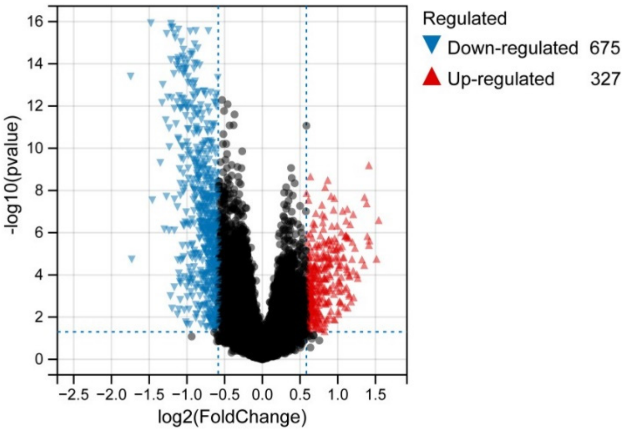

Figure S4 Volcano plot presenting the DEGs in the TCGA-LUAD cohort based on median M2/M1 ratios.

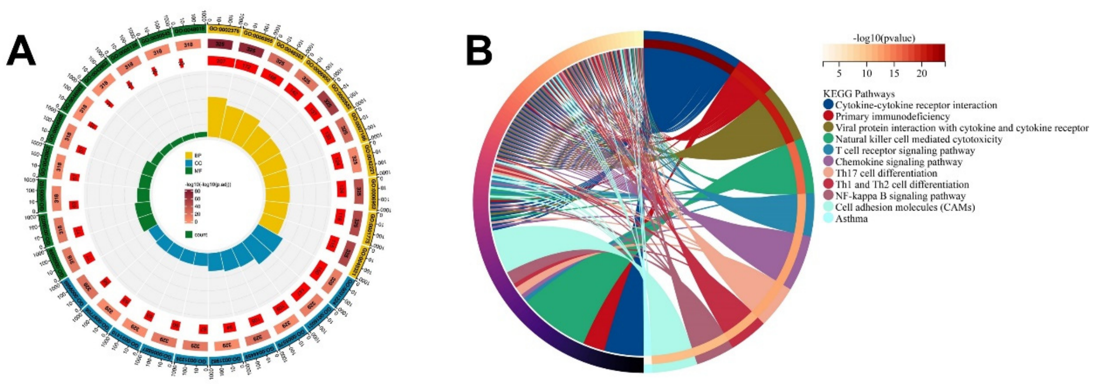

Figure S5 GO and KEGG analysis of DEGs in high and low M2/M1 ratio groups.

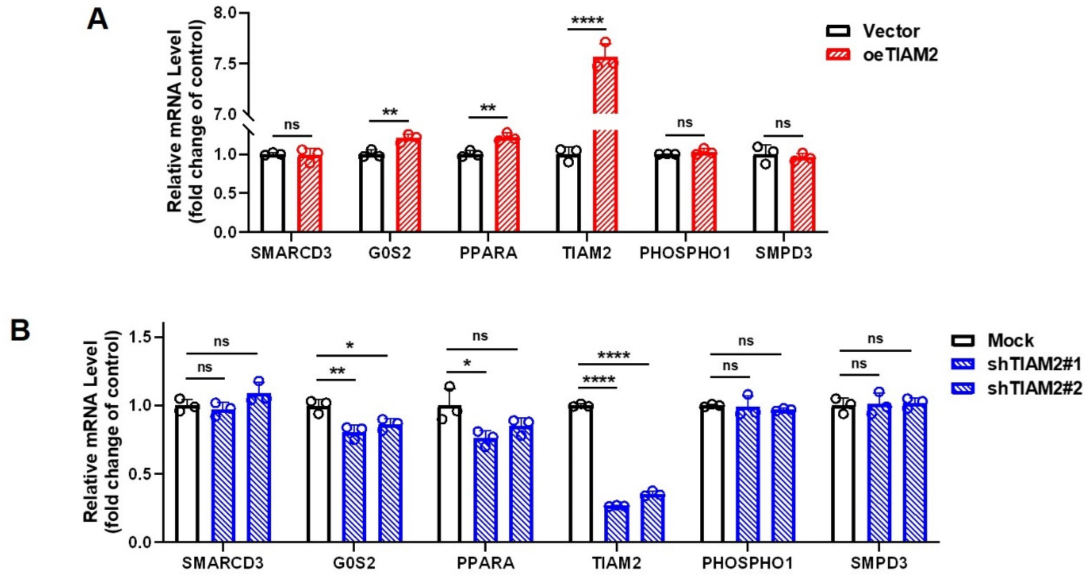

Figure S6 The mRNA levels of 6 lipid metabolism-related genes were detected by RT-qPCR in cell lines overexpressing or knockdown *TIAM2*.

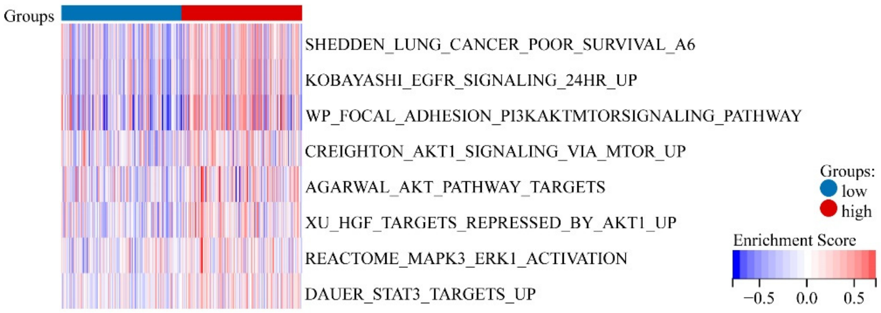

Figure S7 GSVA analysis on samples from high-risk and low-risk groups of TCGA.

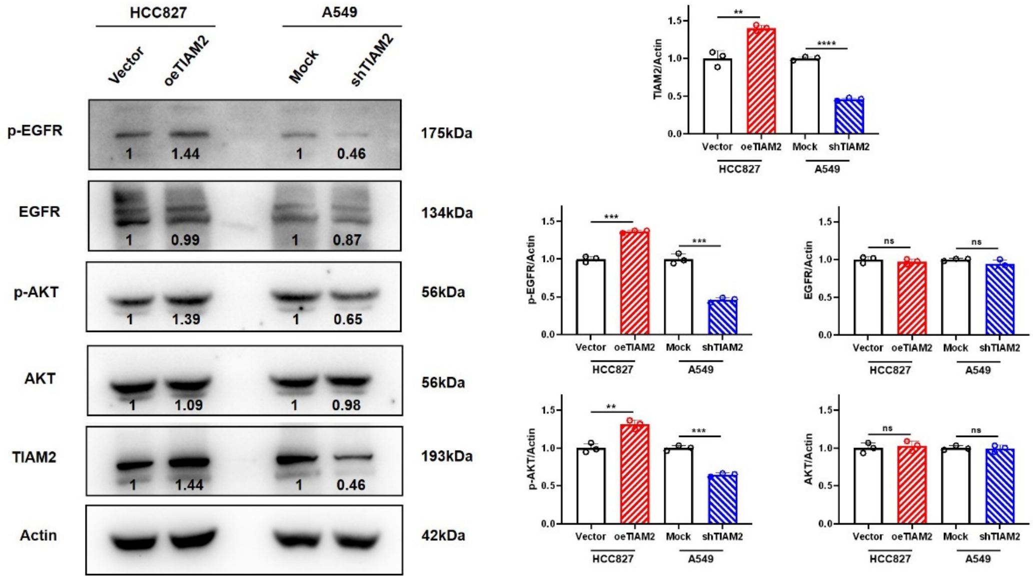

Figure S8 Left, protein expression levels of p-EGFR, EGFR, p-AKT and AKT in overexpressing or knockdown cell lines were detected by WB. Right, amounts of p-EGFR, EGFR, p-AKT, AKT and TIAM2 were quantified from experiment.
